# Dietary L-Tryptophan Modulates the Hematological Immune and Antibacterial Ability of the Chinese Mitten Crab, *Eriocheir sinensis*, Under Cheliped Autotomy Stress

**DOI:** 10.3389/fimmu.2018.02744

**Published:** 2018-12-06

**Authors:** Cong Zhang, Qian Zhang, Xiaozhe Song, Yangyang Pang, Yameng Song, Yongxu Cheng, Xiaozhen Yang

**Affiliations:** ^1^Demonstration Center for Experimental Fisheries Science Education, Shanghai Ocean University Shanghai, China; ^2^Key Laboratory of Freshwater Aquatic Genetic Resources, Ministry of Agriculture, Shanghai Ocean University Shanghai, China; ^3^National Engineering Research Center of Aquaculture, Shanghai Ocean University Shanghai, China

**Keywords:** *Eriocheir sinensis*, autotomy cheliped, L-tryptophan, hematological immune, antioxidant capacity

## Abstract

In pond cultures of juvenile *Eriocheir sinensis*, limb autotomy stress seriously affects and restricts the quality and economic benefits of aquaculture. This study was designed to evaluate the effects of dietary supplementation of L-tryptophan on *E. sinensis* under the cheliped autotomy stress. In the present study, 252 crabs were divided into four groups: dietary L-trp supplementation with 0.28, 0.40, 0.53, and 0.70%, and their hematological immunity, antioxidant capacity, anti-stress, and antibacterial ability were evaluated after 14 days of using biochemical analysis, flow cytometry, and molecular biology techniques. First, we counted the mortality after 14 days of feeding and found that compared with other treatments, dietary supplementation of 0.53 and 0.70% L-trp significantly lowered the mortality of *E. sinensis*. Moreover, the total hemocyte count (THC), hemocyanin, and glutathione (GSH) content, and glutathione peroxidase (GSH-Px) activity significantly increased at 7 and 14 d with dietary supplementation of 0.53 and 0.70% L-trp, in contrast with the significant decrease in malondialdehyde (MDA) content at 14 d in the same dietary groups (*P*<*0.05*). Next, the bacterial challenge test after 14 days of feeding showed that the THC levels, phagocytic rate, and acid phosphatase (ACP) and alkaline phosphatase (ALP) activity were significantly higher with dietary supplementation of 0.53 and 0.70% L-trp after 12 and 24 h of *Aeromonas hydrophila* injection, along with a significant improvement in the antioxidant capacity (*P*<*0.05*). Further, we measured the expression of antibacterial-related protein genes (*EslecB* and *HSP 90*) and found that they were significant up-regulated in the hepatopancreas, hemocytes, intestine, and gill in the groups with dietary supplementation of 0.53% and 0.70% L-trp after 12 h or 24 h of *A. hydrophila* injection (*P*<*0.05*). Taken together, the observations in this study indicate that dietary supplementation of L-trp can enhance the antioxidant capacity and improve the hematological immune status and antibacterial ability of *E. sinensis* under the cheliped autotomy stress, thereby increasing the survival rate of *E. sinensis* under cheliped autotomy stress.

## Introduction

The Chinese mitten crab, *Eriocheir sinensis*, occupies an important position in China's aquaculture industry, owing to its rich nutritional value and wide market demand. However, in pond cultures of *E. sinensis*, various factors, such as fighting, defense, and foraging, can cause a high rate of limb autotomy, (1–4). Limb autotomy has many adverse effects on the crabs, including long-term loss of function and energy (5), as well as, decreased feeding efficiency and survival rate (6, 7). Moreover, limb autotomy reduces the ability of immune defense to resist pathogens (8). Zhao et al. reported that coin-sized crabs have up to 30% limb autotomy rate in earthen pond culture conditions of *E. sinensis* (9). The problem of limb autotomy stress has seriously affected and restricted the quality and aquaculture economic benefits of *E. sinensis*, resulting in widespread concern (8, 10, 11).

Several studies have shown that nutritional adjustment can regulate the immune system of aquatic animals and is one of the effective means to enhance anti-stress ability (12, 13). As an important part of animal diet, amino acids play an important role in the growth and immune regulation of aquatic animals (12, 14). Among them, tryptophan as an essential amino acid can improve the inflammation response (15), which can be used as feed grade at present. Studies have reported that dietary supplementation with tryptophan can modulate intestinal immune response and antioxidant status in *Ctenopharyngodon Idella* (16) and regulate the non-specific immune response in *Apostichopus japonicus Selenka* (17), which play an important role in immune regulation and anti-stress responses in aquatic animals.

At present, agricultural activities have changed the natural balance between pairs of original hosts and their pathogens, which could lead to the emergence of diseases and other serious problems for the aquaculture industry (15, 18). Pathogenic *Aeromonas hydrophila* can cause serious diseases such as “Tremble Disease” and “Edema Disease” in *E. sinensis* (19). Therefore, *A. hydrophila* can be used as an experimental infection bacterium to evaluate the antibacterial ability of *E. sinensis*.

Crustaceans lack acquired immune system and their immune system mainly includes hematological and cellular immunity. Hematological immunity further includes some humoral immune factors present in the hemolymph, such as heat shock proteins 90 (HSP 90) (20), C-type lectin (21), hemocyanin (22), and some immune-related enzymes such as hydrolases (23) and, antioxidant enzymes (24). Hemocyte immunity mainly includes phagocytosis, package action, agglutination, and melanization of hemocytes (25). In invertebrates, hemocyte phagocytosis is widely used to assess their antibacterial ability (26). When invertebrates are attacked by pathogens, oxygen free radicals are released to enhance their antibacterial ability (27). In addition, hemocytes can adhere to pathogens, trigger phagocytosis, and produce highly toxic reactive oxygen species (ROS) (28). Our previous studies have shown that melatonin (N-acetyl-5-methoxytryptamine) can significantly improve the serum antioxidant capacity of *E. sinensis* (29). As the precursor of melatonin, tryptophan is an effective scavenger for free radicals and can maintain the cellular redox balance by enhancing the body's antioxidant capacity (30). Moreover, many studies have reported that hemocyanin, HSP 90, and C-type lectins *EslecB* play important roles in the anti-stress response and immune defense response against pathogen attacks (20–22).

Therefore, dietary supplementation of key amino acids is an effective means of improving animal immunity, which is a more cost-effective and safer solution than one involving adding antibiotics (12). However, to date, there is no report on the effects of dietary supplementation of L-trp on the anti-stress and antibacterial ability of *E. sinensis*. Therefore, this study was designed to evaluate the effects of dietary supplementation of L-trp on hematological immunity, antioxidant capacity, andanti-stress and antibacterial ability of *E. sinensis*, in order to provide some scientific guidance for improving the anti-stress and disease resistance of *E. sinensis* from a nutritional perspective.

## Materials and Methods

### Diets

The composition and nutritional level of the basal diet is presented in Table 1. The main protein sources of feed were rapeseed meal, soybean meal and cotton meal, while the fat sources were pork lard, fish oil, and phosphatide oil. Based on studies on *Scylla serrata* and *Astacus leptodactylus* (31–33), the L-trp contents in the four experimental diets in this study were determined to be 0.28 % (Diet # A) (control), 0.40% (Diet # B), 0.53% (Diet # C), and 0.70% (Diet # D), respectively. L-trp (≥99.7%) was purchased from Sinopharm Chemical Reagent Co., Ltd (China). Ingredients were ground into fine powder through a 187.5 μm mesh sieve. Then weigh accurately, using a step-by-step expansion method to add trace L-tryptophan, mix evenly, and use a double screw extruder to make pellet feed at 1.5 mm diameter. Then spread out and dried in an oven at 55°C. After cooling under natural conditions, it was stored in a ziplock bag and stored in a refrigerator at −20°C.The actual content of L-trp in different diets was determined by reversed-phase high-performance liquid chromatography (RP-HPLC). A C18 (μ–Bondapak Cl8 column, diameter 25 cm × 4.6 mm) column was selected, the mobile phase was composed of sodium acetate buffer + methanol = 95 + 5, the flow rate was 1.5 mL/min, ultraviolet (UV) detection wavelength was 280 nm, the injection volume was 15 μL, and the column was at room temperature.

**Table 1:** Ingredients and proximate composition of the control diets (% dry matter).

**Ingredient**	**Content**
Soybean meal	15.50
Peanut meal	8.00
Rapeseed meal	18.00
Cotton meal	7.00
Fish meal	7.00
Wheat flour	28.30
Yeast meal	2.00
Squid powder	2.00
Phosphatide oil	2.00
Fish oil	1.50
Pork lard	1.50
Mineral mix^a^	0.30
Vitamin mix^b^	1.20
Ca(H_2_PO_4_)_2_	1.00
Choline chloride	0.40
Dishulin	0.10
Bentonite	4.00
Salt	0.20
Total	100.00
**ANALYZED COMPOSITION**	
Moisture	11.45
Crude protein	34.56
Crude lipid	8.34
Ash	9.15

a *Vitamin premix (per kg diet): vitamin A, 62500 IU; vitamin D_3_, 15000 IU; vitamin E, 1.75 g; vitamin K_3_, 35.4 mg; vitamin B_1_, 100 mg; vitamin B_2_, 150 mg; vitamin B_6_, 150 mg; vitamin B_12_, 0.2 mg; biotin, 4 mg; D-calcium pantothenate, 250 mg; folic acid, 25 mg; nicotinamide, 300 mg; vitamin C, 700 mg*.

b *Mineral premix (per kg diet): FeSO_4_·H_2_O, 200 mg; CuSO_4_·_5_H_2_O, 96 mg; ZnSO_4_·H_2_O, 360 mg; MnSO_4_·H_2_O, 120 mg; MgSO_4_·H_2_O, 240 mg; KH_2_PO_4_, 4.2 g; NaH_2_PO_4_, 0.5 g; KI, 5.4 mg; CoCl_2_·_6_H_2_O, 2.1 mg; Na_2_SeO_3_, 3 mg*.

### Experimental Crabs

All experimental protocols were reviewed and approved by the Animal Bioethics Committee, Shanghai Ocean University, China. In May 2018, 350 hard-shelled crabs that had just finished molting and limb-intact *E. sinensis* (*Crustacea*; *Decapoda*; *Grapsidae*) juvenile crabs (16.89 ± 3.87 g), were collected from an earth pond at the Chongming research base of Shanghai Ocean University (Shanghai, China). Juvenile crabs were acclimated in 24-L ultra-clear glass tanks, each of which was supplied with continuous aerated freshwater at 24–28°C, pH 7.84 ± 0.08, DO concentration 6.3 ± 0.4 mg/L, salinity 0.3%, total ammonia 0.36 ± 0.03 mg/L, chloride level 136 ± 15 mg/L, and basal nitrite < 0.05 mg/L^−1^ and natural photoperiod conditioning for 1 week. The crabs were fed once a day with a commercial crab diet (Diet # A).

### Experimental Design

**Figure 1:**
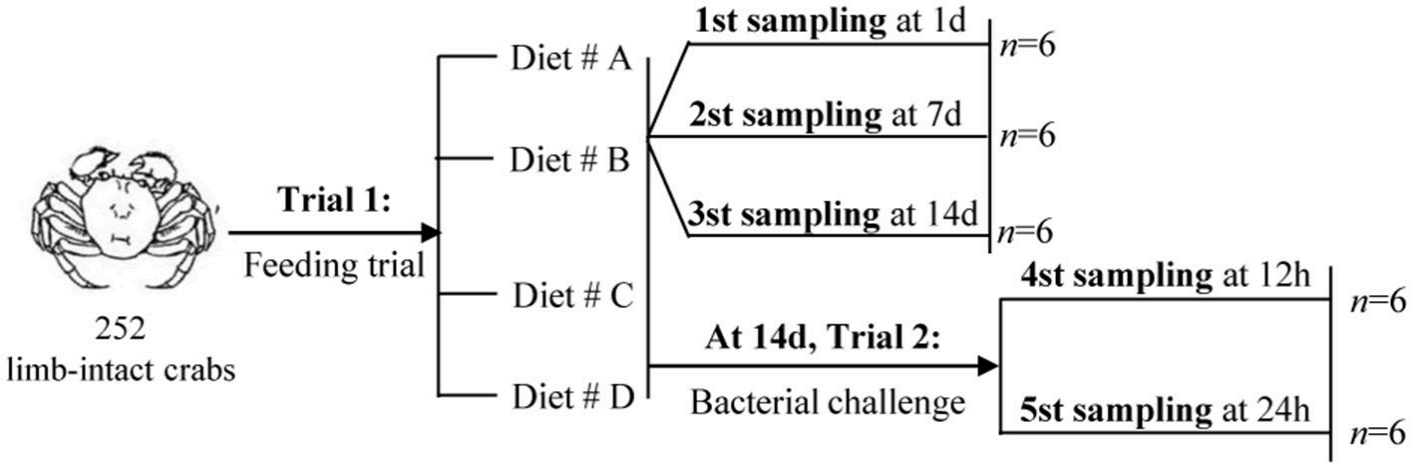
The experimental design and sampling procedures.

The experimental design and sampling procedures are shown in Figure 1. A total of 252 limb-intact crabs were selected from the above samples and subjected to induction for autotomy of the left cheliped. For this, the researchers gently grasped the limbs of the crabs using their fingers, and the crab would spontaneously autotomize the corresponding limbs. Next, all the autotomized crabs were randomly divided into four groups: Diet # A, Diet # B, Diet # C, and Diet # D. Each diet group had three replicates. The crabs were returned to the aerated water in monoculture systems immediately afterwards, and maintained under the environmental conditions described above.

#### Trial 1: Feeding Trial

A previous study had reported that 14 days of feeding on diets supplemented with individual amino acids are enough to modulate physiological and immune responses in aquatic animal (34). The feeding trial in this study lasted for 2 weeks, with an aim to evaluate the effects of short-term dietary supplementation with L-trp on cellular and hematological immune status. The mortality of all groups was calculated at the end of the experiment. Hemolymph were collected at 1, 7, and 14 d, respectively, since the start of the experiment. Hemolymph was drawn using a sterile 1-ml syringe from the unsclerotized membrane of the right third periopod and was diluted 1:1 with steriled anticoagulation mixture (trisodium citrate 30 mM, NaCl 338 mM, glucose 115 mM, EDTA 10 mM). The mixture was centrifugated at 42,000 × g for 5 min to separate the serum and the hemocytes, and then stored at −20°C for evaluation of THC levels, hemocyanin content and antioxidant capacity.

#### Trial 2: Bacterial Challenge Test

This experiment was designed to investigate the effect of L-trp supplementation on the antibacterial ability and immunomodulation after bacterial infection of *E. sinensis*. At the end of the *Trial 1* (after 14 days of feeding), the bacterial challenge test was carried out. Frozen sample of *Aeromonas hydrophila* was obtained from Shanghai Ocean University (8). The cultured bacteria were resuspended in crustacean saline (NaCl 0.21M, KCl 13.6 mM, H_3_BO_3_ 8.6 mM, NaOH 4.75 mM, MgSO_4_ j 7H_2_O 20 mM, pH 7.2), and the concentration of the suspension was adjusted to 4 × 10^5^ CFU / mL (the Median lethal concentration (LC_50_) obtained from pre-experimental analysis) (8). Each crab was injected with 100 μL bacterial suspension. Crabs were sampled for hepatopancreas, hemolymph, gill and intestine collection at 12 and 24 h after *A. hydrophila* injection. Hemolymph was collected and centrifuged as described above, and the serum and hemocytes were then collected separately for further experimental analysis.

### Hemolymph Samples Analysis

#### Hemocyte Level of THC

The levels of THC were obtained with a drop of the anti-coagulant hemolymph placed in a hemocytometer using a Leica DMIL microscope (Leica Microsystems GmbH, Wetzlar, Germany) and each crab count was repeated three times.

#### Immune-Related Parameters

Hemocyanin concentrations were determined by a UV-Spectrophotometric (Beijing Purkinje General Instrument Co., Ltd) at 335 nm with 10 μL of serum diluted in 990 μL distilled water in a quartz cuvette, manually calibrated with distilled water. Hemocyanin concentrations (mmol/L) = 2.69 E (1%, 1 cm) mmol/L (35).

The acid phosphatase (ACP), alkaline phosphatase (ALP) were measured by a UV-spectrophotometer (Beijing Purkinje General Instrument Co., Ltd) at 520 nm with corresponding detection kits (Nanjing Jiancheng Bioengineering Institute, Nanjing, China) according to the manufacturer's protocols.

#### Anti-Oxidant Defense Systems Parameters

Commercial kits obtained for SOD, GSH, GSH-Px, and MDA from Nanjing Jiancheng Bioengineering Institute (Nanjing, China) were used to measure their activities in the hemolymph supernatant. They were measured using a UV-spectrophotometer (Beijing Purkinje General Instrument Co., Ltd) at 520, 420, 412, and 532 nm as described by the manufacturer's protocols, respectively.

#### Hemocyte Phagocytosis

The hemocyte phagocytosis was analyzed by using a BD Accuri™ C6 flow cytometer (BD Biosciences, USA). In trial 2, the hemocyte collected after centrifugation were resuspended in 0.1 M PBS buffer (NaCl 136.89 mM; KCl 2.67 mM; Na_2_HPO_4_ 8.10 mM; KH_2_PO_4_ 76 mM; pH 7.2–7.4) (Sangon Biotech Co., Ltd., Shanghai, China). Thirty microliter fluorescent microspheres mother liquor (FluoSpheres^TM^ Carboxylate-Modified Microspheres, 1.0 μm, red fluorescent, 580/605, F881, Invitrogen) were added to 1.5 ml of PBS buffer and mixed to prepare a fluorescent microspheres suspension. Transfer 200 μl of blood cell suspension into 1.5 ml EP tube, add 50 μl of fluorescent microspheres suspension, mix well, and avoid light reaction at 18°C for 1 h. The reaction was stopped by the addition of 250 μl of Baker's formol fixative (4% formaldehyde, 2% NaCl) and then sequentially determined by flow cytometry. Each sample analysis included a total of 2,00,00 events and the flow speed was maintained at < 300 s^−1^. Phagocytosis was defined as the proportion of hemocytes that had ingested at least three fluorescent beads. The data were analyzed by using the BD CellQuest™ Pro software (BD Biosciences, USA).

### Expression of the HSP 90 Gene Level: Quantitative RT-PCR

Total RNA was extracted from the hemocyte, hepatopancreas intestinal and gill tissues using RNAiso^TM^ plus reagent (RNA Extraction Kit, TaKaRa, Japan) according to the manufacturer's protocol. The concentration and quality of the total RNA were estimated by micro-volume ultraviolet-visible spectrophotometer (Quawell Q5000; Thmorgan, China) and agarose-gel electrophoresis, respectively, and reverse transcribed with the PrimeScript™ RT reagent Kit (Perfect Real Time, TaKaRa, Japan) according to the manufacturer's protocol. The obtained cDNA that was diluted to 1:2 with double-distilled water was used as qRT-PCR template. Relative quantification was performed using the ABI 7,500 Real-Time PCR System (Life Technology, USA) with a ChamQ™ Universal SYBR^®;^ qPCR Master Mix (Vazyme Biotech Co.,Ltd, Nanjing, China) kits using the following program: 95°C for 30 s; 40 cycles at 95°C for 5 s, 60°C for 34 s; followed by a melting curve at 95°C for 15 s, 60°C for 1 min, 95°C for 15 s. The PCR primer sequences for *HSP 90* is shown in Table 2 (Sangon Biotech Co., Ltd., Shanghai, China). β*-actin* was used as the internal control and performed in triplicate for every sample. Relative changes in gene expression levels were determined by 2^−ΔΔ*Ct*^ method. Data were analyzed and presented as average values ± standard deviation (SD), as well as, the n-fold difference relative to the control data.

**Table 2:** Primer information for quantitative real-time polymerase chain reaction.

**Primers**	**Sequences (5^′^-3^′^)**	**Usage**
*EsLecB-F*	GACAGGCATCAACGAGAAGGA	Real-time -PCR
*EsLecB-R*	CACAGTTGTAAGTTATTGTATCCCG	Real-time -PCR
*HSP 90-F*	GAAGGTGATCCGCAAGAACC	Real-time -PCR
*HSP 90-R*	GTTGGTGGAGTCCTCATGGA	Real-time -PCR
*β-actin -F*	TCATCACCATCGGCAATGA	Real-time -PCR
*β-actin -R*	TTGTAAGTGGTCTCGTGGATG	Real-time -PCR

### Statistical Analyses

Data are presented as the average values of six individuals ± standard deviation (SD) (*n* = 6), before the test, each sample was an independent individual and no pooling was carried out. The percentage values (dependent variable) were arcsine transformed before analysis. The effects of treatment were statistically analyzed using an analysis of variance (one-way ANOVA, LSD and Duncan analysis), and a *P* < 0.05 was considered significant. All statistical analyses were performed using SPSS 20.0 software (Chicago, USA; Version 22.0).

## Results

### Mortality and Hemolymph Analysis After Dietary Supplementation of L-TRP for 14d

#### Mortality

**Figure 2:**
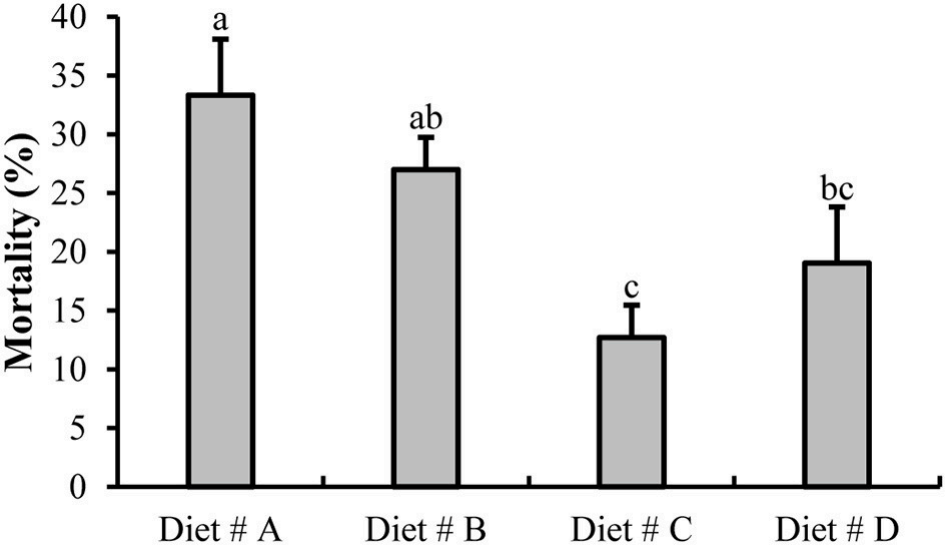
The mortality of *E. sinensis* for all groups after fed dietary treatments at 14 d. The values are expressed as the means ± SD (*n* = 21). Different letters placed above the column represent the significant differences (*P*<*0.05*).

At the end of the experiment at 14 d, we evaluated the mortality of all groups as shown in Figure 2. The mortality of crabs in Diet # C (12.70 ± 2.75%) and Diet # D (19.05 ± 4.76%) groups were significantly lower than that in the control group (Diet # A group) (33.33 ± 4.76 %) (*P*<*0.05*). The mortality of Diet # C group exhibited the lowest value among all other groups. The results showed that dietary supplementation of L-trp can significantly reduce the mortality of *E. sinensis*.

#### THC Levels and Hemocyanin Content

**Figure 3:**
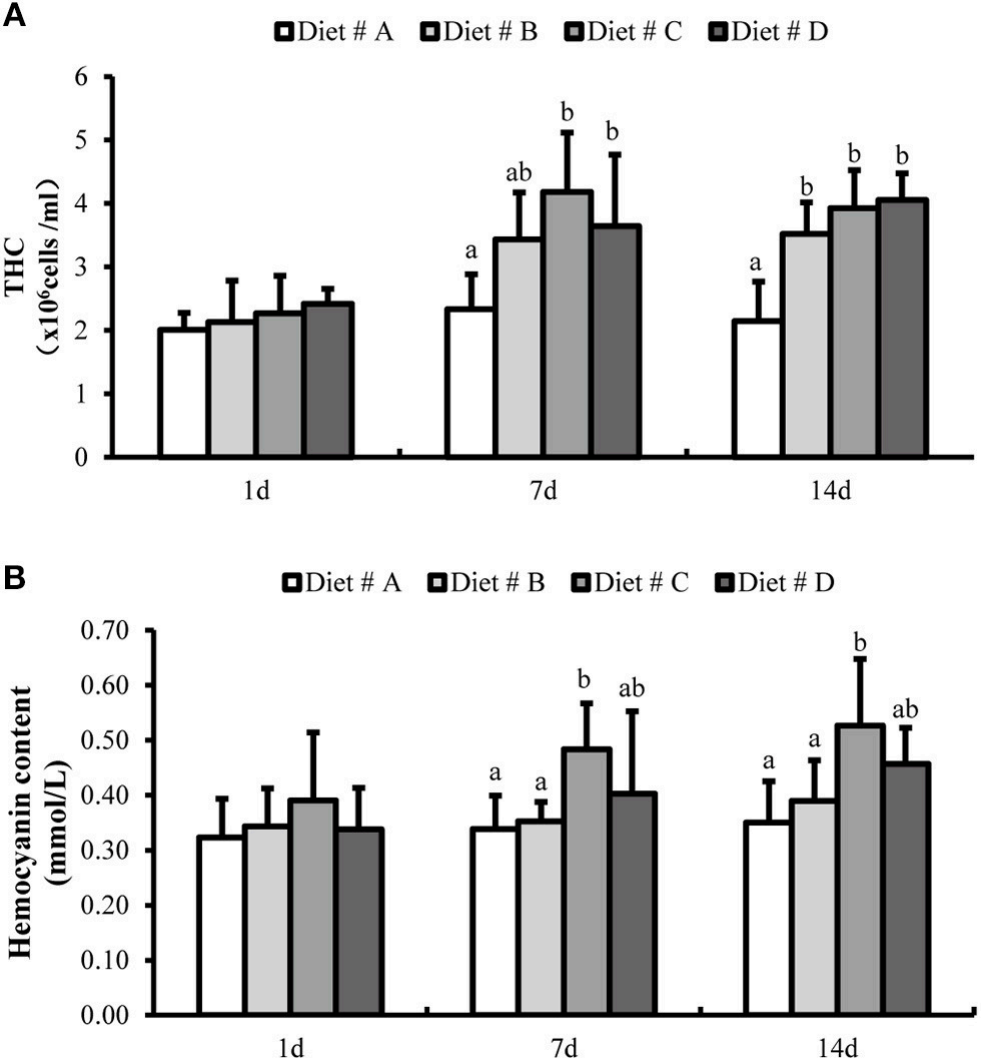
The total hemocyte counts (THC) **(A)** and hemocyanin contents **(B)** in *E. sinensis* with different treatment at 1, 7, and 14 d. The values are expressed as the means ± SD (*n* = 6). Different letters placed above the column represent the significant differences between different groups at the same time (*P*<*0.05*).

There was no significant difference in the THC levels and hemocyanin content among the four dietary groups at 1 d after the start of the experiment (Figure 3). However, the THC levels were significantly higher in the L-trp supplement groups than in the Diet # A group (*P*<*0.05*) at 7 and 14 d, whereas there the THC levels was no significant difference among between the three L-trp supplement groups (Figure 3A). The hemocyanin content in Diet # C and Diet # D groups was significantly higher than that in Diet # A group at 7 d and 14 d (*P*<*0.05*), with the highest level observed in Diet # C group (Figure 3B).

#### Antioxidant Capacity

There was no significant difference in SOD activity among the four diet groups (Table 3). The GSH contents were significantly higher in Diet # C group at 7 d and in Diet # D group at 14 d than in Diet # A group (*P*<*0.05*) (Table 3). The GSH-Px activity tended to gradually increase with the increased L-trp supplementation in diet, and it was significantly higher in Diet # D than in other groups at 7 d (*P*<*0.05*) (Table 3). The MDA content was significantly lower in Diet # C and Diet # D groups than in Diet # A group at 14 d (*P*<*0.05*) (Table 3). The results showed that dietary supplementation of L-trp significantly enhanced the serum antioxidant capacity of *E. sinensis*.

**Table 3:** Effect of L-trp supplement on the serum antioxidant capacity in *E. sinensis* at 1, 7, and 14 d.

**Item**	**Sample time**	**Diet # A**	**Diet # B**	**Diet # C**	**Diet # D**
SOD activity (U/ml)	1 d	82.90 ± 7.08	79.54 ± 8.16	81.84 ± 6.03	77.24 ± 4.89
	7 d	85.66 ± 6.55	86.78 ± 3.78	83.43 ± 7.58	84.54 ± 8.68
	14 d	88.72 ± 4.77	95.79 ± 4.45	89.61 ± 10.98	97.20 ± 2.50
GSH content (mg/L)	1 d	1.06 ± 0.27	1.20 ± 0.34	1.23 ± 0.37	1.25 ± 0.50
	7 d	0.98 ± 0.28^a^	1.17 ± 0.38^a^	1.85 ± 0.41^b^	1.44 ± 0.42^ab^
	14 d	1.28 ± 0.39^a^	1.47 ± 0.37^ab^	1.44 ± 0.27^ab^	1.87 ± 0.22^b^
GSH-Px activity (μmol/L)	1 d	424.96 ± 24.63	414.52 ± 43.04	441.39 ± 28.15	411.91 ± 55.13
	7 d	408.78 ± 65.04^a^	413.74 ± 47.67^a^	441.91 ± 28.97^a^	535.30 ± 26.00^b^
	14 d	371.22 ± 12.99	387.91 ± 62.76	433.30 ± 35.83	387.39 ± 88.46
MDA content (nmol/ml)	1 d	12.26 ± 2.26	12.64 ± 2.93	12.03 ± 2.64	11.31 ± 2.67
	7 d	12.34 ± 2.47	11.46 ± 2.19	9.76 ± 1.21	11.05 ± 2.02
	14 d	11.58 ± 2.69^a^	11.05 ± 2.18^ab^	7.98 ± 1.88^b^	7.34 ± 1.69^b^

*The values are expressed as the means ± SD (n = 6). SOD: superoxide dismutase; GSH, glutathione; GSH-Px, glutathione peroxidase; MDA, malondialdehyde. Different letters represent the significant differences between different groups at the same time (P < 0.05)*.

### Bacterial Challenge Test

#### Hematological Immune Status

**Figure 4:**
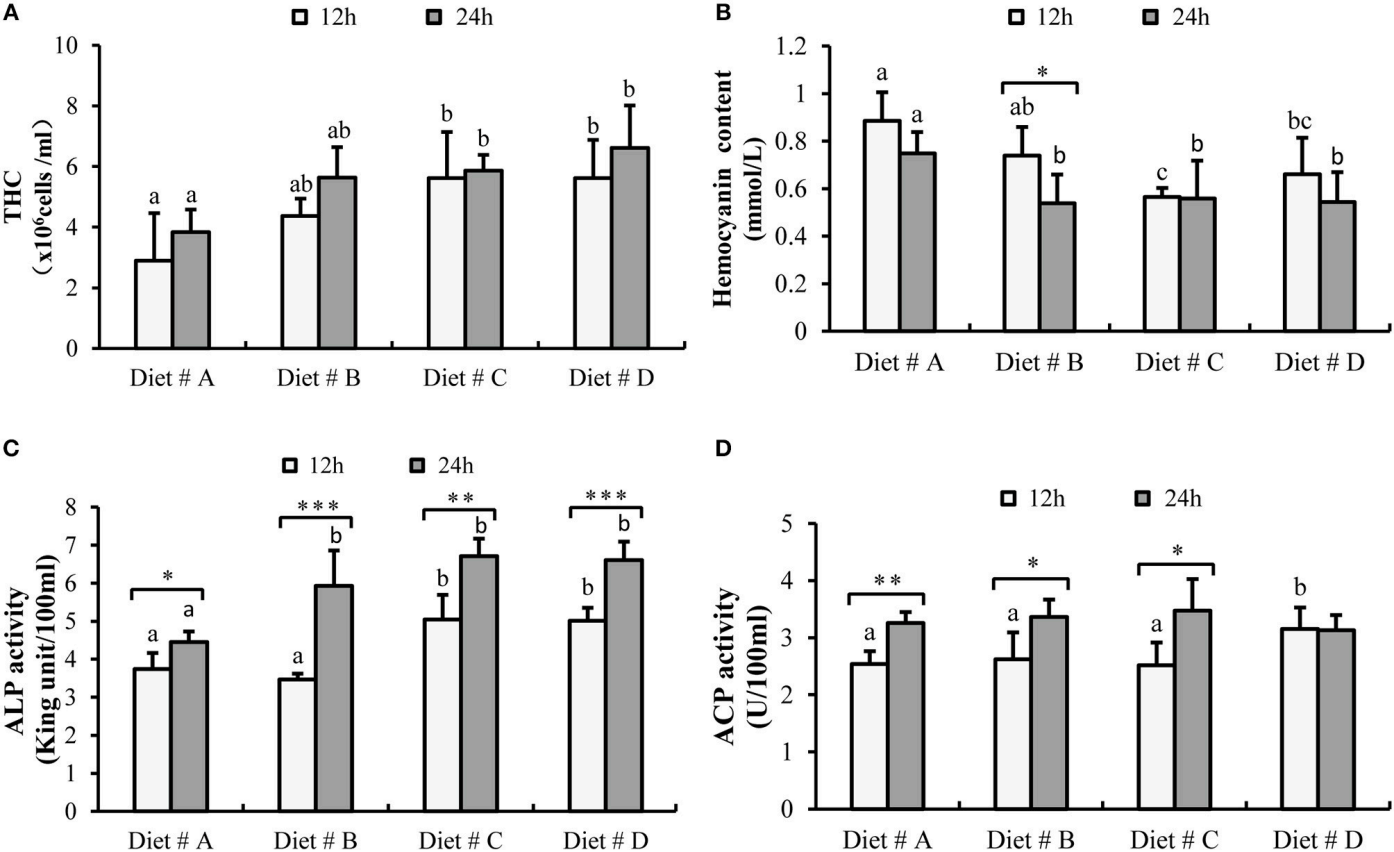
The hematological immune status of *E. sinensis* fed dietary treatments at 12 and 24h after *A. hydrophila* injection. **(A)** THC: total hemocyte counts; **(B)** Hemocyanin content; **(C)** ACP: acid phosphatase; **(D)** ALP: alkaline phosphatase. The values are expressed as the means ± SD (*n* = 6). Different letters placed above the column represent the significant differences between different groups at the same time (*P*<*0.05*).

The THC levels in Diet # C and Diet # D group were significantly higher than those in the control group after 12 and 24h of *A. hydrophila* injection (*P*<*0.05*) (Figure 4A).

**Figure 5:**
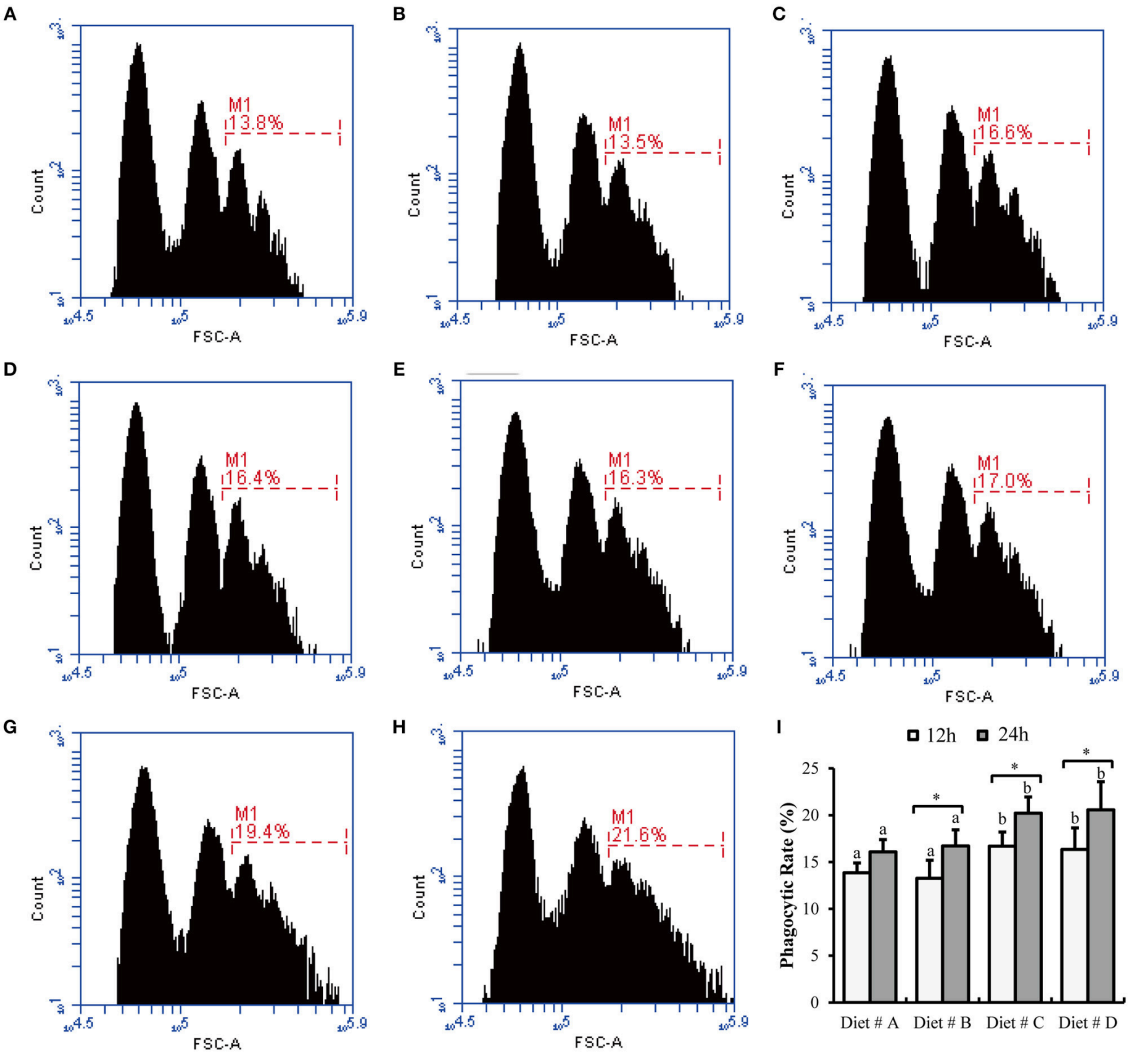
The hemocytes phagocytic rate of *E. sinensis* fed dietary treatments at 12 and 24 h after *A. hydrophila* injection. **(A)** 12 h- Diet # A; **(B)** 12 h- Diet # B; **(C)** 12h- Diet # C; **(D)** 12 h- Diet # D; **(E)** 24 h- Diet # A; **(F)** 24 h- Diet # B; **(G)** 24 h- Diet # C; **(H)** 24 h- Diet # D; **(I)** phagocytic rate. The values are expressed as the means ± SD (*n* = 6). Different letters placed above the column represent the significant differences between different groups at the same time (*P*<*0.05*).

Some representative images of hemocyte phagocytosis obtained by flow cytometry are shown in Figures 5A–H. The phagocytic rate of hemocyte in Diet # C and Diet # D group was significantly higher than that in the control group after 12 and 24 h of *A. hydrophila* injection and a significant increased was observed at 24 h compared with 12 h after *A. hydrophila* injection in Diet # B, Diet # C and Diet # D group (*P*<*0.01*) (Figure 5I).

The hemocyanin content in Diet # C group and Diet # D group was significantly lower than that in the control group after 12 and 24 h of *A. hydrophila* injection (*P*<*0.05*) and it was significant decreased at 24 h compared with 12 h after *A. hydrophila* injection in Diet # B group (*P*<*0.05*) (Figure 4B).

The ACP activity was significant higher in Diet # D group than that in the other three diet groups (*P*<*0.05*) at 12 h after *A. hydrophila* injection and it was significant increased at 24 h compared with 12 h after *A. hydrophila* injection in Diet # A (*P*<*0.01*), Diet # B (*P*<*0.05*) and Diet # C (*P*<*0.05*) groups (Figure 4C).

The ALP activity was significantly higher in Diet # C and Diet # D group at 12 h and it was significantly higher in Diet # B, Diet # C and Diet # D at 24 h than in the control group after *A. hydrophila* injection (*P*<*0.05*) (Figure 4D). Moreover, the ALP activity was significantly increased at 24 h compared with 12 h after *A. hydrophila* injection in all diet groups (*P*<*0.05, P*<*0.001, P*<*0.01, P*<*0.001*, respectively) (Figure 4D). The results showed that dietary supplementation of L-trp significantly improved the hematological immune status with *A. hydrophila* injection.

#### Serum Antioxidant Capacity

**Figure 6:**
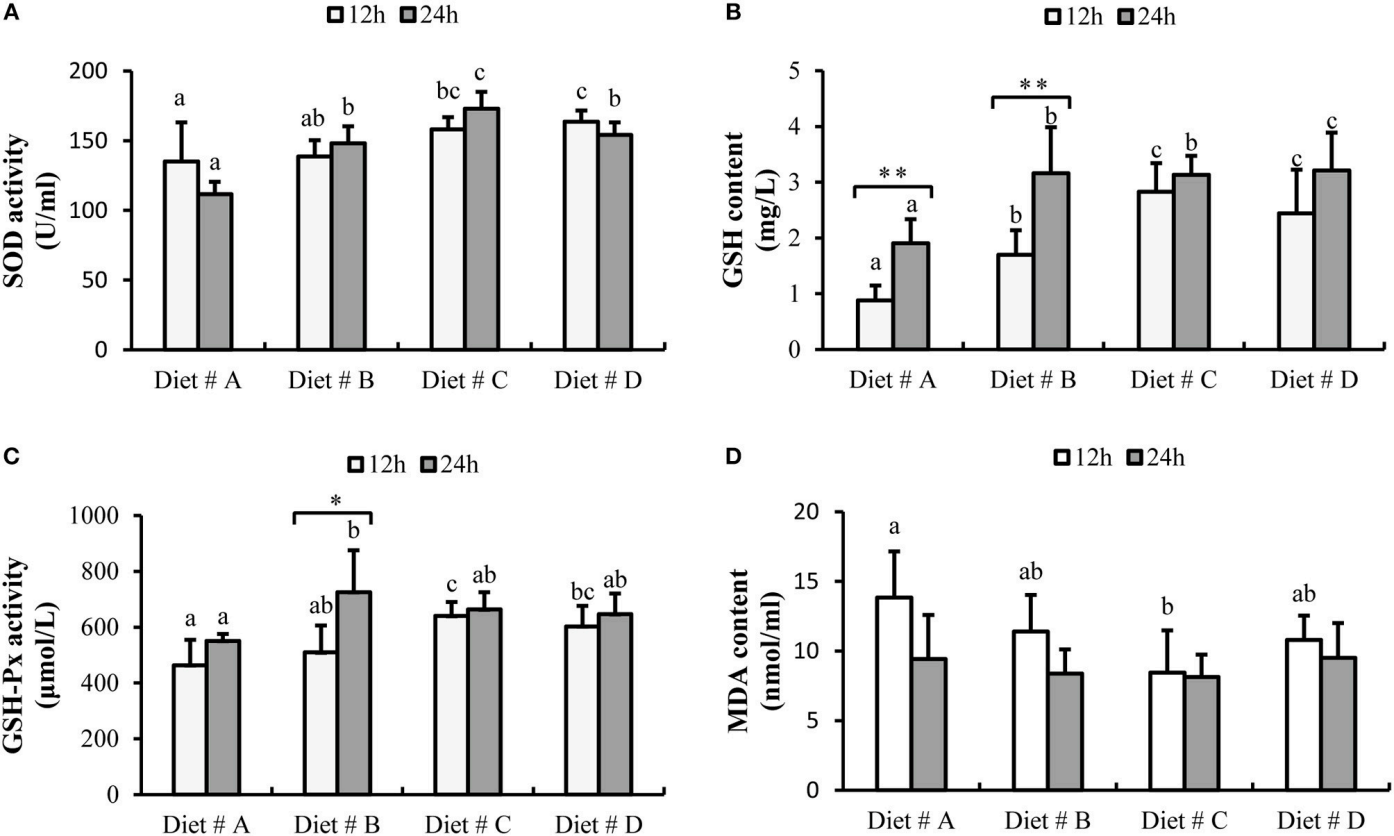
The serum antioxidant capacity of *E. sinensis* fed dietary treatments at 12 and 24 h after *A. hydrophila* injection**. (A)** SOD, superoxide dismutase; **(B)** GSH, glutathione; **(C)** GSH-Px, glutathione peroxidase; **(D)** MDA, malondialdehyde. The values are expressed as the means ± SD (*n* = 6). Different letters placed above the column represent the significant differences between different groups at the same time (*P*<*0.05*). “*” represent significant differences between 12 and 24 h at the same diet group (^*^P<*0.05*, ^**^P<*0.01*).

The SOD activity was significantly increased in Diet # C and Diet # D group at 12 h, and significantly higher in Diet # B, Diet # C and Diet # D at 24 h than in the control group after *A. hydrophila* injection (*P*<*0.05*). Moreover, the maximum level was observed in Diet # C group at 24 h after *A. hydrophila* injection (*P*<*0.05*) (Figure 6A).

The GSH content in Diet # B, Diet # C and Diet # D groups was significantly higher than that in the control group after 12 and 24 h of *A. hydrophila* injection, and the maximum level was observed in Diet # D group at 24 h after *A. hydrophila* injection (*P*<*0.05*). Moreover, the GSH content was significantly increased at 24 h compared with 12 h after *A. hydrophila* injection in Diet # A and Diet # B groups (*P*<*0.01*) (Figure 6B).

The GSH-Px activity was significantly increased in Diet # C and Diet # D groups at 12 h and it was significant higher in Diet # B at 24 h than that in the control group after *A. hydrophila* injection (*P*<*0.05*). Moreover, the GSH-Px activity was significantly increased at 24 h compared with 12 h after *A. hydrophila* injection in Diet # B group (*P*<*0.05*) (Figure 6C).

The MDA content was significantly lower in Diet # C group than in the control group at 12 h after *A. hydrophila* injection (*P*<*0.05*), whereas no significant difference among the four groups was observed at 24 h (Figure 6D). The results showed that dietary supplementation of L-trp significantly enhanced the serum antioxidant capacity of *E. sinensis* that had been injected with *A. hydrophila*.

#### Antibacterial-Related Protein Genes Expressions

**Figure 7:**
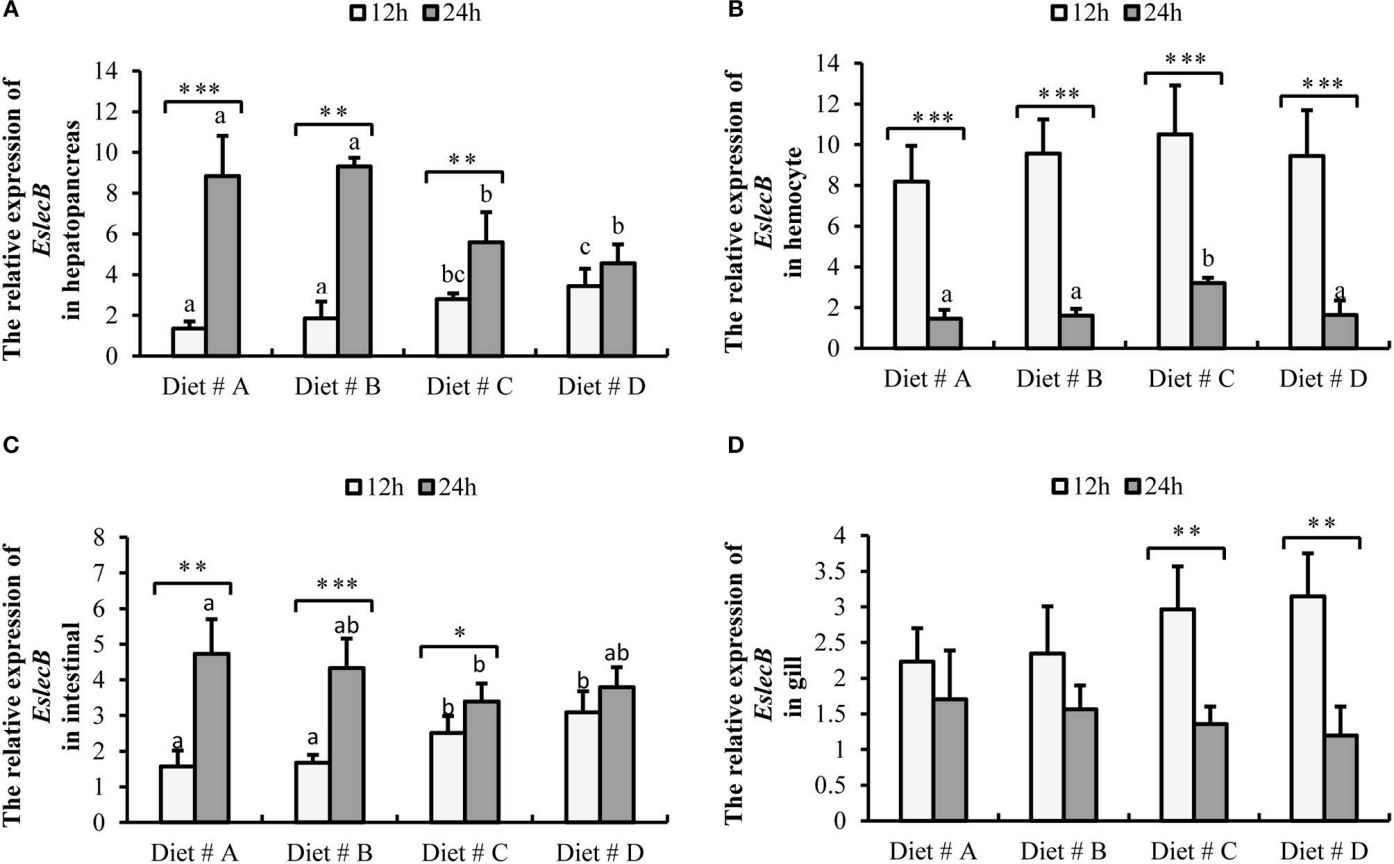
Expression level of *EslecB* gene normalized to β*-actin* in the hepatopancreas, hemocyte, intestinal and gill tissues of *E. sinensis* fed dietary treatments at 12 and 24 h after *A. hydrophila* injection. The values are expressed as the means ± SD (*n* = 4). Different letters placed above the column represent the significant differences (*P*<*0.05*) ^“*”^ represent significant differences between 12 and 24 h at the same diet group (^*^P<*0.05*, ^**^P<*0.01*, ^***^P<*0.001*). **(A)** The *EslecB* relative expression in hepatopancreas; **(B)** The *EslecB* relative expression in hemocyte; **(C)** The *EslecB* relative expression in intestinal; **(D)** The *EslecB* relative expression in gill.

The expression of *EslecB*-mRNA in the hepatopancreas was significantly higher in Diet # C and Diet # D group at 12 h, whereas it was significantly lower in Diet # C and Diet # D group at 24 h than in the other two groups after *A. hydrophila* injection (*P*<*0.05*) (Figure 7A). Moreover, the expression of *EslecB*-mRNA was significantly increased at 24 h compared with 12 h after *A. hydrophila* injection in Diet # A (*P*<*0.001*), Diet # B (*P*<*0.01*), and Diet # C (*P*<*0.05*) groups (Figure 7A).

The expression of *EslecB*-mRNA in hemocytes showed no significant difference among the four groups at 12 h, whereas it was significantly higher in Diet # C group at 24 h than in the other three groups after *A. hydrophila* injection (*P*<*0.05*) (Figure 7B). Moreover, the expression of *EslecB*-mRNA was significantly decreased at 24 h compared with 12 h after *A. hydrophila* injection in Diet # A (*P*<*0.001*), Diet # B (*P*<*0.001*), Diet # C (*P*<*0.001*), and Diet # D (*P*<*0.001*) groups (Figure 7B).

The expression of *EslecB*-mRNA in intestine was significantly higher in Diet # C and Diet # D groups at 12 h, whereas it was significantly lower in Diet # C group at 24 h than in the control group after *A. hydrophila* injection (*P*<*0.05*). Moreover, the expression of *EslecB*-mRNA was significantly increased at 24 h compared with 12 h after *A. hydrophila* injection in Diet # A (*P*<*0.01*), Diet # B (*P*<*0.001*), and Diet # C (*P*<*0.05*) groups (Figure 7C).

The expression of *EslecB*-mRNA in gill showed no significant difference among the four groups at 12 and 24 h after *A. hydrophila* injection. However, the expression of *EslecB*-mRNA was significantly decreased at 24 h compared with 12 h after *A. hydrophila* injection in Diet # C (*P*<*0.01*) and Diet # D (*P*<*0.01*) groups (Figure 7D).

**Figure 8:**
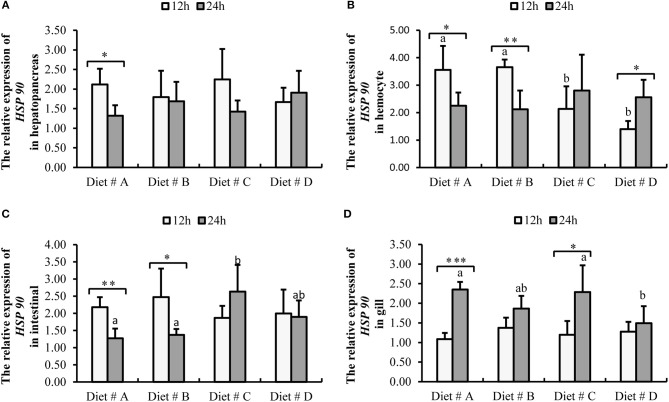
Expression level of *HSP 90* gene normalized to β*-actin* in the hepatopancreas, hemocyte, intestinal and gill tissues of *E. sinensis* fed dietary treatments at 12 and 24 h after *A. hydrophila* injection. The values are expressed as the means ± SD (*n* = 4). Different letters placed above the column represent the significant differences (*P*<*0.05*) ^“*”^ represent significant differences between 12 and 24 h at the same diet group (^*^P<*0.05*, ^**^P<*0.01*, ^***^P<*0.001*). **(A)** The *HSP 90* relative expression in hepatopancreas; **(B)** The *HSP 90* relative expression in hemocyte; **(C)** The *HSP 90* relative expression in intestinal; **(D)** The *HSP 90* relative expression in gill.

The expression of *HSP 90*-mRNA in hepatopancreas showed no significant difference among the four groups at 12 and 24 h after *A. hydrophila* injection. However, the expression of *HSP 90*-mRNA was significantly decreased at 24 h compared with 12 h after *A. hydrophila* injection in Diet # A group (*P*<*0.05*) (Figure 8A).

The expression of *HSP 90*-mRNA in hemocytes was significantly lower in Diet # C and Diet # D groups at 12 h (*P*<*0.05*), whereas there was no significant difference among the four groups at 24 h after *A. hydrophila* injection (Figure 8B). Moreover, the expression of *HSP 90*-mRNA was significantly decreased in Diet # A (*P*<*0.05*) and Diet # B (*P*<*0.01*) groups at 24 h compared with 12 h after *A. hydrophila* injection, whereas it was significantly increased in Diet # C group at 24 h compared with 12 h after *A. hydrophila* injection (*P*<*0.05*) (Figure 8B).

The expression of *HSP 90*-mRNA in intestine was not significantly different among the four groups at 12 h after *A. hydrophila* injection (*P*<*0.05*), whereas it was significantly higher in Diet # C group than in the other three groups. Moreover, the expression of *HSP 90*-mRNA was significantly decreased in Diet # A (*P*<*0.01*) and Diet # B (*P*<*0.05*) groups at 24 h compared with 12 h after *A. hydrophila* injection (Figure 8C).

The expression of *HSP 90*-mRNA in gill showed no significant difference among the four groups at 12 h after *A. hydrophila* injection, whereas it was significantly lower in Diet # D group than in the control group (*P*<*0.05*). Moreover, the expression of *HSP 90*-mRNA was significantly increased in Diet # A (*P*<*0.001*) and Diet # C (*P*<*0.05*) groups at 24 h compared with 12 h after *A. hydrophila* injection (Figure 8D). The results showed that dietary supplementation of L-trp significantly up-regulated the antibacterial-related protein genes expression levels of *E. sinensis* that were injected with *A. hydrophila*. Thus, the antibacterial ability of *E. sinensis* was significantly improved with dietary supplementation of L-trp.

## Discussion

### The Effects of dietary L-trp Supplementation on Hematological Immunity

Crustaceans lack an acquired immune system and only possess an innate immune system, which includes hematological and cellular immunity. Several studies on crustaceans have demonstrated that hematological parameters are important parameters for assessing their immune response ability, such as THC, and hematological immune-related proteins and enzymes (36–38). In this study, we determined the effects of dietary L-trp supplementation on the hematological parameters of left cheliped autotomized *E. sinensis*, as well as, the effects on hematological immunity and disease resistance, THC in crustaceans is a commonly used performance parameter for assessing cellular immunity (39). Hemocyte immunity mainly includes phagocytosis, package action, agglutination, and melanization of hemocyte, and participate in the removal of pathogens (25). Moreover, hemocyte phagocytosis is widely used to assess the antibacterial ability in invertebrates (26). In the present study, we found that dietary L-trp supplementation significantly increased the THC levels in *E. sinensis* at 7 and 14 d. We speculated that the wound is susceptible to pathogens after limb autotomy of *E. sinensis*, and the increase in THC level can accelerate the removal of foreign bodies and substance transport in the body. In trial 2, we found that the THC levels in Diet # C and Diet # D group were significantly higher than those in the control group after 12 and 24 h of *A. hydrophila* injection. Moreover, the phagocytic activity of hemocyte in Diet # C and Diet # D groups was significantly higher than that in the control group after 12 h and 24 h of *A. hydrophila* injection. It indicates that supplementation of L-trp in diet can significantly increase the THC levels and the ability of pathogens removal. (17) reported that supplementation of 3% TRP in diet significantly increased the hemocyte phagocytic activity of *Apostichopus japonicus* Selenka, which was consistent with our results.

Hemocyanin is an important multifunctional protein in crustaceans, that is found mainly in the hemolymph, and accounts for more than 90% of serum total protein (40–42). In addition to the function of carrying oxygen, transporting metal ions, storing protein and regulating osmotic pressure, hemocyanin exhibits antibacterial, antiviral, and phenoloxidase activity under certain conditions, and is an important participant of immune defense system (22, 43). The hemocyanin content in the hemolymph acts as a good indication of the health status of crustaceans (44). In trial 1, we observed a similar trend in hemocyanin contents and THC levels, wherein the hemocyanin content in Diet # C group was significantly higher than that in other diet groups. The results showed that dietary L-trp supplementation can improve the hematological immunity and anti-stress ability of *E. sinensis*, similar to the results of a study on *Apostichopus japonicus* Selenka (17). However, we observed in the results of trial 2 that the hemocyanin content was significantly lower in the L-trp supplement group than in the control group after injection of *A. hydrophila*. Moreover, the hemocyanin content of all the diet groups showed a trend of reduction at 24 h compared with 12 h after *A. hydrophila* injection. We speculate that the hemocyte phagocytic activity, antibacterial ability and ability of foreign bodies body removal in the L-trp supplementation groups were significantly enhanced, resulting in a large consumption of hemocyanin not being timely supplemented. Machado et al. found that the concentration of hemoglobin was significantly lower in the tryptophan supplement group than in the control group in *Dicentrarchus labrax* after infection with *Photobacterium damselae* subsp. *piscicida* (*Phdp*) (15). Qin et al. found a significant increase in THC at 12 h after infection with *A. hydrophila* in *E. sinensis* (45), similar to our results.

When a pathogen is phagocytosed by phagocytic cells, it fuses with lysosomes and is eventually hydrolyzed by various hydrolases. Hydrolases not only exist in cells, but are also widely distributed in the serum by means of degranulation, to form a hydrolase system, which plays an important role in the serum immune defense. They are considered to be important non-specific indicators of crustaceans, as ALP and ACP levels reflect the health status of aquatic animals (23, 46). In the present study, we observed that dietary supplementation with 0.70% L-trp significantly increased the ACP activity at 12 h after *A. hydrophila* injection, and ACP activity was significantly higher at 24 h than that at 12 h in the other three dietary group. ALP activity showed a similar trend. Dietary supplementation of L-trp significantly increased the activity of ACP and ALP in the serum, which was beneficial in accelerating the body metabolism and enhanced the ability of crabs to remove pathogenic bacteria. Previous study reported that dietary supplementation of tryptophan can significantly increase ACP activity in the plasma of *Dicentrarchus labrax* (15). Christophermarlowea et al. reported that the ALP activity of *Gadus morhua* L. was increased after exposure to a crowding stress during the latter part of the post-stress period (47). In this study, dietary supplementation of L-trp could improve the resistance to pathogens in crabs to some extent.

### The Effects of Dietary L-trp Supplementation on Hemolymph Antioxidant Capacity

When invertebrates are attacked by foreign pathogens, oxygen is released to enhance resistance to prevent infection with pathogens (27, 28). Therefore, the antioxidant system is an important immune defense system for crustaceans. In addition, hemocyte can adhere to pathogens, trigger phagocytosis, and produce highly toxic reactive oxygen species (ROS) (48). Various antioxidant enzymes, oxidases and hydrolases play important roles before phagocytosis, during phagocytosis, and after phagocytosis. In crustaceans, the production of ROS is an important indicator of cell defense (27). ROS is indispensable for normal cell functions (such as redox signals and anti-pathogens), but excessive ROS can cause oxidative damage to tissues, such as oxidative damage of DNA, cell membranes, proteins, and enzymes (49). In order to prevent oxidative damage to the organism by excessive ROS, the antioxidant defense system gets activated and removes excess ROS. Superoxide dismutase (SOD), glutathione peroxidase (GSH-Px), and glutathione (GSH) are important members of the crustacean antioxidant defense system (24). In the present study, although dietary supplementation of L-trp had no significant effect on SOD activity, the GSH content with dietary supplementation of 0.53% L-trp at 7 d 0.70% L-trp at 14 d was significantly higher than that of the control group. In the group with dietary supplementation 0.70% L-trp, GSH-Px activity at 7 d was significantly higher than that in other groups. Moreover, we observed that dietary supplementation with L-trp significantly increased the serum antioxidant capacity of *E. sinensis* at 12 and 24 h after infection with *A. hydrophila*. Studies have reported that L-trp can increase the SOD activity of *Apostichopus japonicus* Selenka (17). Mardones et al. reported that dietary tryptophan significantly reduced cortisol levels in the plasma of *Salmo salar* and *Oncorhynchus kisutch*, enhancing their anti-stress ability (50). In rats, a lack of Trp in the diet leads to a decrease in GSH-Px activity in liver tissue. In rats, a lack of tryptophan in the diet leads to a decrease in GSH-Px activity in liver tissue (51). As the main decomposition product of lipid peroxidation, MDA can reflect the degree of lipid peroxidation in the body and the degree of oxidative damage in cells (52). In this study, dietary supplementation with 0.53 and 0.70% L-trp significantly reduced the serum MDA levels at 14 d. The MDA levels in dietary supplementation groups of 0.53% L-trp and 0.70% L-trp were significantly lower than those in control group at 12 and 24 h after infection with *A. hydrophila*. This result indicated that dietary supplementation of proper L-trp can inhibit lipid peroxidation in *E. sinensis*. Niyogi et al. reported that MDA levels were negatively correlated with antioxidant enzyme activity (53). Previous studies have shown that tryptophan can reduce MDA levels in rat liver (54). Wen et al. reported that dietary supplementation of tryptophan significantly reduced the MDA levels in the gut of *Ctenopharyngodon idella*, and significantly increased the SOD and GSH-Px activity, as well as, GSH content (16), which is consistent with our results.

### The Effects of Dietary L-trp Supplementation on Gene Expression of Anti-bacterial-Related Protein

In invertebrates, C-type lectin can participate in pathogen recognition and binding, agglutination, antibacterial, hemocyte encapsulation, activation of prophenoloxidase (proPO) activation system and other immune responses (55–59). Many studies have reported that some C-type lectins in *E. sinensis*, such as *EsLecA, EslecG, EslecD*, and *EslecF*, can promote hemocyte encapsulation and antibacterial activity in antibacterial reactions (60–62). In addition, it has been reported that C-type lectin *immulectin-2* has the effect of inducing phagocytosis in Manduca sexta (59). As a congenital immune-related gene, C-type lectin *EslecB* participates in immune defense responses such as microbial binding, cell agglutination, and defense against bacterial attack in *E. sinensis* (21). The hemocytes, hepatopancreas, and gills are considered the important tissues involved in immunity of crustaceans. Hemocytes are involved in the recognition and phagocytosis of pathogenic bacteria. Hepatopancreas is responsible for hematopoiesis, immunity, detoxification, digestion, and other physiological functions. As an important respiratory organ and excretory organ, gill can isolate the body from the surrounding microorganisms, effectively avoid infection, and resist the invasion of pathogenic bacteria (25, 58, 63). As a complex micro-ecological system, the intestines have the dual functions of digestion, absorption and disease defense (64). Therefore, in this study, we used qRT-PCR to detect the expression of antibacterial-related protein genes *EsLecB* and *HSP 90* in hepatopancreas, hemocytes, gill and intestine of *E. sinensis* after injection of *A. hydrophila*. The results showed that the expression of *EsLecB* gene in the hepatopancreas, hemocytes, and gill was significantly up-regulated at 12 h after the injection of *A. hydrophila* in the dietary supplementation group with 0.53% L-trp. This indicates that dietary supplementation with L-trp accelerated the antibacterial and immune defense responses. Moreover, the expression of *EsLecB* gene in hemocytes and intestine was significantly lower at 24 than at 12 h after the injection of *A. hydrophila*, whereas the expression of *EsLecB* gene in hepatopancreas and gill was significantly higher at 24 h than at 12 h after the injection of *A. hydrophila* in all diet groups. The results showed that, in order to resist the attack of *A. hydrophila*, hemocytes and intestine are the primary agents of antibacterial defense function in the early stage, which may be related to the induction of hemocyte phagocytosis. Thereafter, the hepatopancreas, and gill act together act as the main functional unit to exert immune defense function.

Heat shock protein HSP 90 is an important disease-resistant and anti-inverse factor in animals. It is an important molecular chaperone, and plays an important role in resisting the invasion of pathogens, and regulating immune function and anti-aging (20). Studies have found that when *Charybdis japonica* was exposed to disrupting chemicals (EDCs), such as bisphenol A (BPA) and 4-nonylphenol (NP), the expression of the *HSP 90* gene in crab tissue was significantly increased in a short time (65). In this study, we found that the *HSP 90* gene expression in the hepatopancreas, intestine, and gill was no significantly difference among the four diet groups at 12 h after the injection of *A. hydrophila*, whereas the *HSP 90* gene expression in the intestine was significantly up-regulated at 24 h after the injection of *A. hydrophila* in Diet # C group compared with the control group. However, the *HSP 90* gene expression in gill was significant down-regulated at 24 h after the injection of *A. hydrophila* in Diet # D group compared with the control group. After the infection of *A. hydrophila* in *E. sinensis*, the expression of *HSP 90* gene varied across different tissues, which may be related to the divergent functions of different tissues in the immune defense system. Our previous study found that eyestalk ablation could lead to a significant up-regulation of *HSP 90* gene expression in hemocytes to improve the body's anti-stress response (11). In the present study, the expression of *HSP 90* gene in hemocytes was significantly lower in Diet # C and Diet # D groups than in the control group at 12 h after the injection of *A. hydrophila*, whereas there was no significant difference among the four groups at 24 h after the injection of *A. hydrophila*. The results showed that dietary supplementation of L-trp can enhance the body's anti-stress ability to a certain extent in a short period of time.

## Conclusion

In summary, dietary supplementation of L-trp can enhance the antioxidant capacity, improve the hematological immune status, and increase the survival rate of *E. sinensis* under cheliped autotomy stress. Moreover, the bacterial challenge test results showed that dietary supplementation of L-trp can enhance the immune defense against bacterial attack by regulating the hemocyte phagocytosis, hydrolase and antioxidant defense systems, and expression of antibacterial-related protein genes. This study evaluated the effects of dietary supplementation of L-trp on the hematological immune, antioxidant capacity, anti-stress, and antibacterial ability of *E. sinensis*, which can provide scientific guidance for improving the anti-stress and disease resistance of *E. sinensis* from the perspective of nutrition.

## Data Availability

The data underlying this study can be found in Data Sheet 1 in the Supplementary Material.

## Author Contributions

CZ designed the experiment and wrote the article. QZ determined the hematological immune parameters. XS determined the expression level of antibacterial-related protein genes. YP and YS assisted in collecting samples. YC provided funding support. XY guided the experiment design and the writing of the article.

### Conflict of Interest Statement

The authors declare that the research was conducted in the absence of any commercial or financial relationships that could be construed as a potential conflict of interest.
